# Development of anti-thrombotic vaccine against human S100A9 in rhesus monkey

**DOI:** 10.1038/s41598-021-91153-y

**Published:** 2021-06-01

**Authors:** Munehisa Shimamura, Koichi Kaikita, Hironori Nakagami, Tomohiro Kawano, Nan Ju, Hiroki Hayashi, Ryo Nakamaru, Shota Yoshida, Tsutomu Sasaki, Hideki Mochizuki, Kenichi Tsujita, Ryuichi Morishita

**Affiliations:** 1 Department of Neurology, Graduate School of Medicine, Osaka University, Suita, Osaka Japan; 2 Department of Health Development and Medicine, Graduate School of Medicine, Osaka University, Suita, Osaka Japan; 3 Department of Cardiovascular Medicine, Graduate School of Medical Sciences, Kumamoto University, Kumamoto, Japan; 4 Department of Clinical Gene Therapy, Graduate School of Medicine, Center of Medical Innovation and Translational Research, Osaka University, 6th floor, Room 0611B, 2-2 Yamada-oka, Suita, Osaka 565-0871 Japan; 5 Department of Neurology, Graduate School of Medicine, Osaka University, 6th floor, Room 0612B, 2-2 Yamada-oka, Suita, Osaka 565-0871 Japan; 6 Department of Health Development and Medicine, Graduate School of Medicine, Osaka University, 6th floor, Room 0612B, 2-2 Yamada-oka, Suita, Osaka 565-0871 Japan

**Keywords:** Stroke, Diseases, Vascular diseases

## Abstract

In post-stroke patients, a decreased adherence to antiplatelet drugs is a major challenge in the prevention of recurrent stroke. Previously, we reported an antiplatelet vaccine against S100A9 in mice, but the use of Freund’s adjuvant and the difference in amino acid sequences in epitopes between mice and humans were problematic for clinical use. Here, we redesigned the S100A9 vaccine for the common sequence in both humans and monkeys and examined its effects in cynomolgus monkeys with Alum adjuvant. First, we assessed several candidate epitopes and selected 102 to 112 amino acids as the suitable epitope, which could produce antibodies. When this peptide vaccine was intradermally injected into 4 cynomolgus monkeys with Alum, the antibody against human S100A9 was successfully produced. Anti-thrombotic effects were shown in two monkeys in a mixture of vaccinated serum and fresh whole blood from another cynomolgus monkey. Additionally, the anti-thrombotic effects were partially inhibited by the epitope peptide, indicating the feasibility of neutralizing anti-thrombotic effects of produced antibodies. Prolongation of bleeding time was not observed in vaccinated monkeys. Although further studies on increasing the effect of vaccine and safety are necessary, this vaccine will be a promising approach to improve adherence to antiplatelet drugs in clinical settings.

## Introduction

Although good adherence to anti-thrombotic agents is crucial in the secondary prevention of ischemic stroke, some patients discontinue these and induce recurrent strokes. For example, approximately 40% of patients diagnosed with recurrent ischemic stroke reportedly discontinued their anti-thrombotic medication^1^. In Sweden, 36.3% of patients were revealed to have discontinued the antiplatelet therapy within 2 years^2^. In the United States, 22.5% of aspirin-treated patients reportedly discontinued aspirin within 1 year and 35.8% of clopidogrel-treated patients stopped treatment^3,4^. Based on this background, we speculated that an anti-thrombotic vaccine could prevent stoke recurrence caused by poor adherence to medication through its long-lasting effects. However, the major problem with anti-thrombotic vaccines is the long-term risk of bleeding and harmful auto-immune response. To resolve this problem, we focused on S100 calcium-binding protein A9 (S100A9), which promotes thrombus formation via secretion from activated platelets and binding to CD36 in platelet itself^5^. In S100A9^−/−^ mice, thrombus formation was reportedly prevented without affecting hemostatic parameters^5^. Although the mechanism of anti-thrombotic effects without increasing the risk of bleeding has not been clarified, the platelet from S100A9^−/−^ mice showed less GpIIb/IIIa activation on collagen under flow conditions^5^. Based on this finding, we have developed an S100A9 vaccine, which could produce antibodies against S100A9 and induce long-term anti-thrombotic effects without affecting bleeding time^6^. This vaccine is a KLH-conjugated B-cell epitope vaccine, including 104 to 113 amino acid sequence at the C-terminus of S100A9, which could avoid harmful T cell responses against self-S100A9 and Th1 predominant immune response^6^. However, differences in amino acid sequence between mice and humans, where the homology is 31.2%, and not using clinically available adjuvant, that is Freund’s adjuvant, were major obstacles for clinical use.

Accordingly, we extended our study to monkeys. We explored the immunogenic epitope of S100A9 in monkeys, which is identical to human sequences, with a clinically available adjuvant, aluminum salt Alum. Next, the anti-thrombotic effects of the produced antibodies were examined using an automated microchip flow-chamber system (total thrombus formation analysis system: T-TAS). Moreover, we examined the reversal effects of epitope peptide for anti-thrombotic effects to clarify whether the produced antibody specifically recognizes the epitope.

## Results

### Screening for appropriate epitope in mice

**Figure 1:**
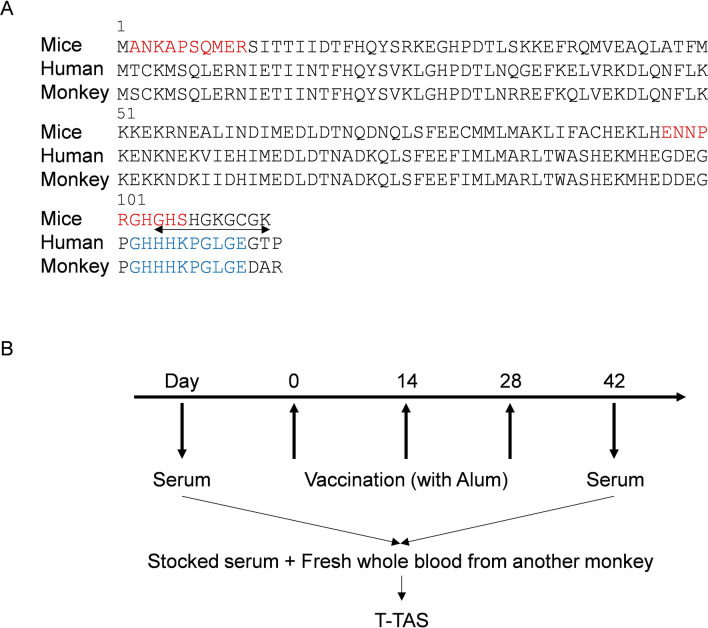
Selection of epitope and study protocol. (**A**) Selected epitope in S100A9 in mice, monkeys, and humans. For screening in mice, new epitopes (in red) and the previous epitope (double arrow)^6^ were examined. The epitope colored blue was used in the monkey experiment. (**B**) Study protocol. Serum is collected before vaccination and 14 days after vaccination. Collected serum was added to the fresh whole blood from another monkey or human, and the mixture was analyzed in T-TAS. *T-TAS* total thrombus formation analysis system.

**Figure 2:**
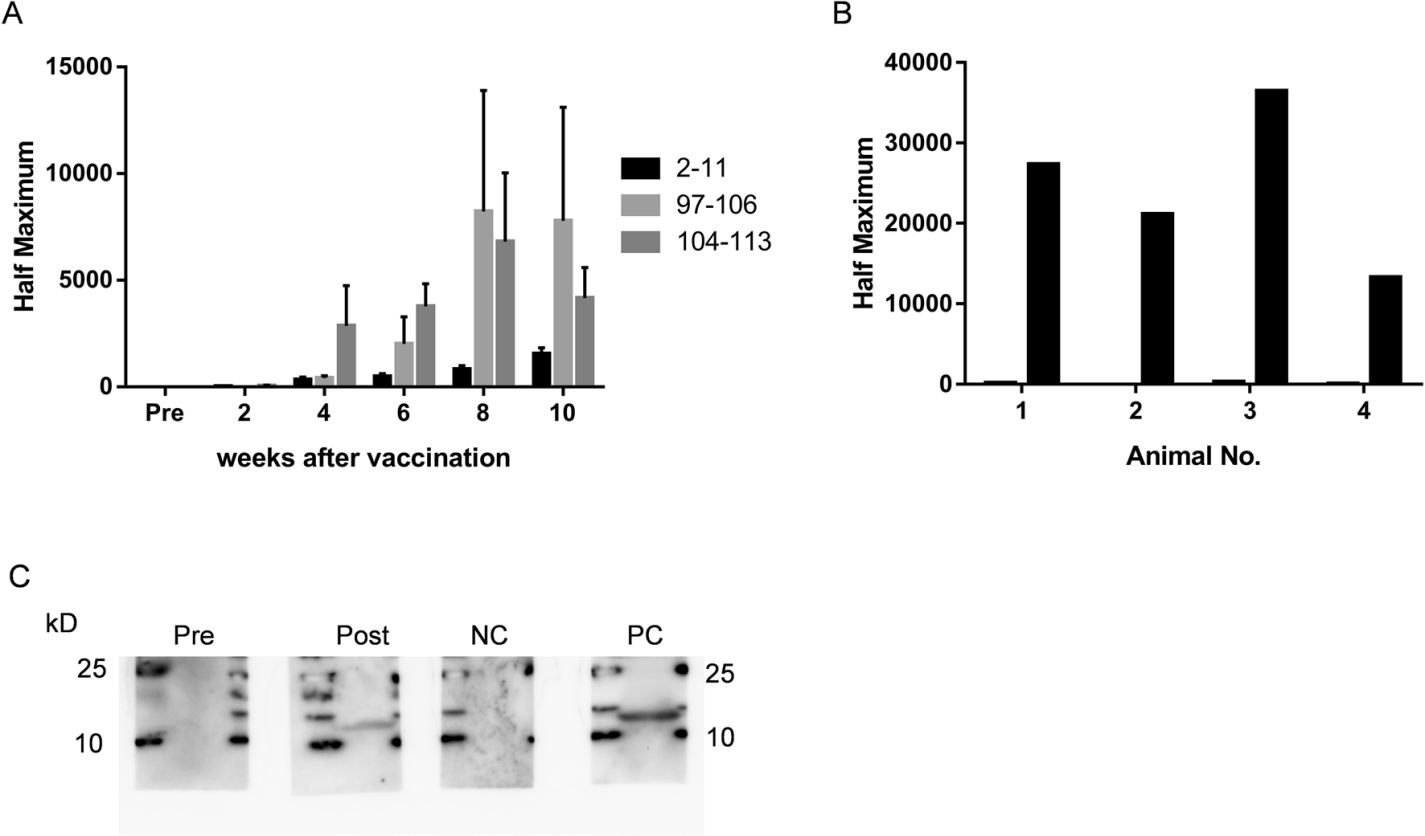
Production of antibody against S100A9 by vaccination. (**A,B**) The antibody titers against the epitope in mice (**A**) and monkeys (**B**). (**A**) The epitopes in C-terminus (97–106 aa and 104–113 aa) are more effective than that in N-terminus (2–11 aa). Data represent the mean ± SEM of n = 3 in each group. (**B**) Antibody titers before (left bar) and after (right bar) the vaccine are presented. All monkeys were successfully vaccinated. (**C**) Western blot analysis for production of antibodies specific for recombinant human S100A9 protein (14 kDa) in No. 4 monkey. The serum samples from pre-vaccination (Pre) and post-vaccination (Post) were examined to determine whether they included antibodies that recognize recombinant S100A9 protein. The commercially available anti-S100A9 antibody (PC) or IgG purified from another normal monkey (NC) was used as a positive control or a negative control, respectively.

Previously, we have selected 104 to 113 amino acids as epitopes in mice according to the 3-dimensional structure and hydrophilicity^6^, but it was unclear whether other epitopes were appropriate. Based on results of computed epitope prediction (Bepipred Linear Epitope Prediction), we first assessed whether other candidate epitopes, 2 to 11 or 97–106 amino acids, where the amino acid sequence is approximately similar between humans and monkeys, could raise antibodies against S100A9 in mice (Fig. 1A). Compared with the vaccine for the 2 to 11 amino acids epitopes, 97–106 amino acids and 104 to 113 amino acids demonstrated higher antigenicity (Fig. 2A), indicating that epitopes in the C-terminus are stronger than those in the N-terminus. Based on this result, we selected 102 to 112 amino acids as the epitope for the S100A9 vaccine, wherein the epitope is consistent between monkeys and humans (Fig. 1A). These peptides were conjugated to KLH, and monkeys were vaccinated 3 times at 14 days intervals (Fig. 1B). The antibody titers, calculated as half maximum (OD50%), against human S100A9 peptide increased 2 weeks after the final vaccination (Fig. 2B). Western blot analysis revealed that the antibodies produced in the serum sample of the vaccinated monkey recognized the recombinant human S100A9 proteins (Fig. 2C, Supplementary Fig. S1).

### Anti-thrombotic effects of S100A9 vaccine

**Figure 3:**
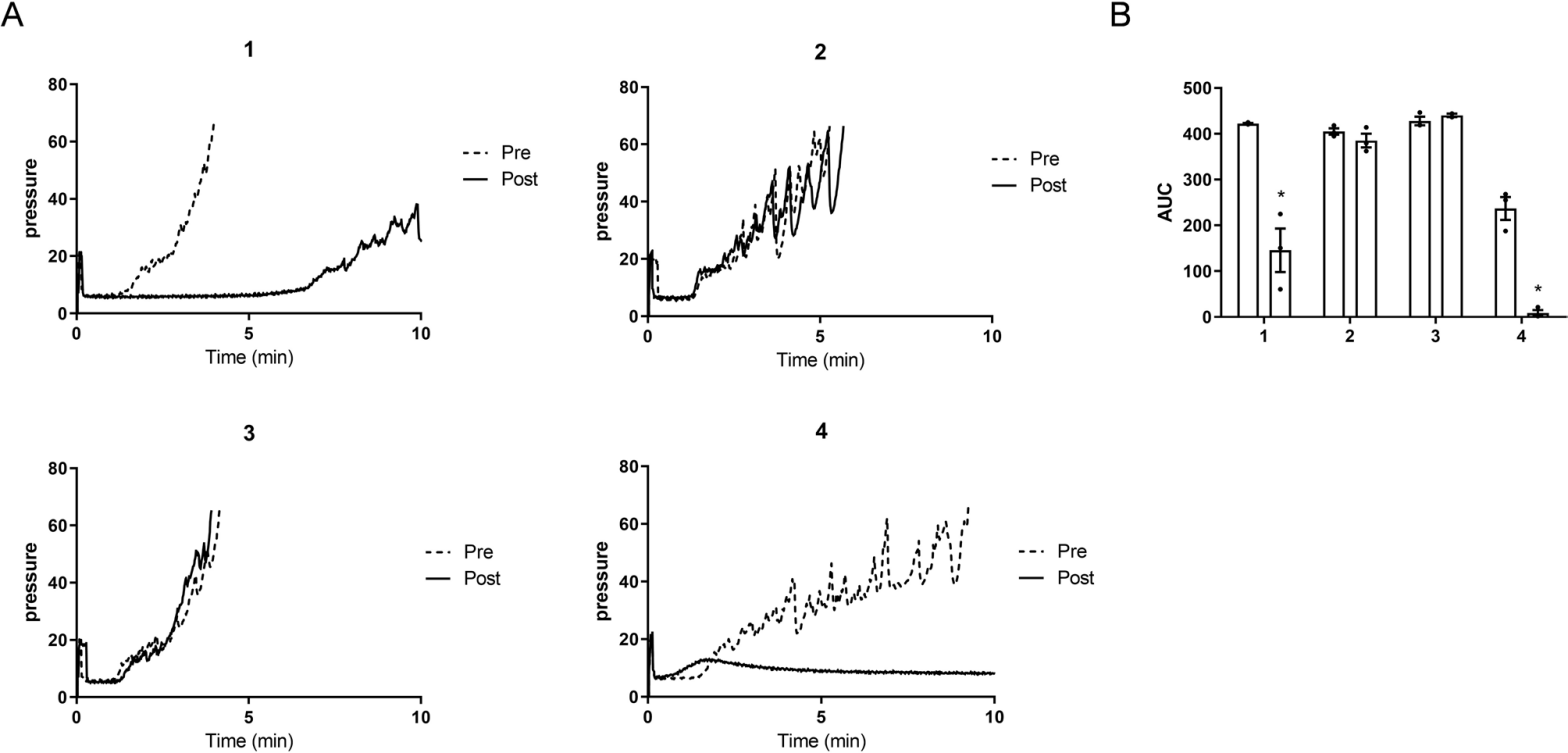
Effect of S100A9 vaccination on thrombus formation. (**A**) Thrombus formation in the mixture of fresh plasma and pre-vaccinated (dotted line) and post-vaccinated (line) serum was evaluated under laminar flow with collagen (T-TAS). (**B**) The AUC in (**A**) was calculated. The post-vaccinated serum from monkeys No. 1 and No. 4 shows a delay in thrombus formation. Data represent the mean ± SEM of 3 times measurements in each group. *Mann–Whitney test, p < 0.05 *vs.* pre-vaccinated monkey. T-TAS, total thrombus formation analysis system.

**Figure 4:**
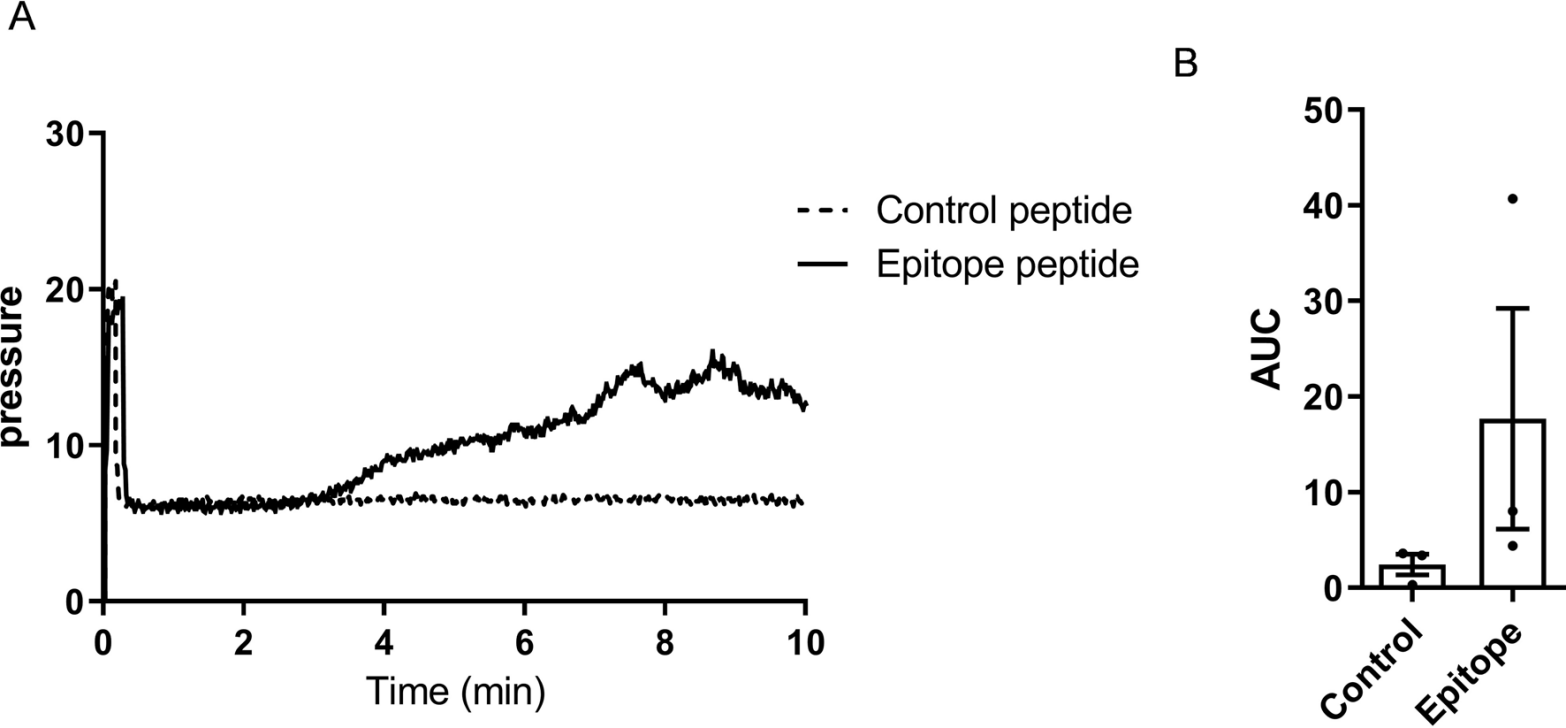
Reversal anti-thrombotic effects by epitope peptide. (**A**) Thrombus formation in the mixture of fresh plasma, post-vaccinated serum from No. 4 monkey, and epitope peptide (line), which could be recognized by the produced antibody. As a negative control, control peptide was added instead of epitope peptide (dotted line). Control peptide had no influences on the anti-thrombotic effects of the peptide vaccine, whereas epitope peptide reversed the anti-thrombotic effect. (**B**) The AUC in (**A**) was calculated. Data represent the mean ± SEM of 3 times measurements. Although there was no significant difference, there was a tendency of increased AUC in the serum mixed with an epitope peptide. *Control* control peptide, *Epitope* epitope peptide.

Next, we examined whether the produced antibody would show anti-thrombotic effects in monkeys using T-TAS. To simultaneously assess the anti-thrombotic effects of the pre-vaccinated and post-vaccinated serum, we stocked the serum at both timings and examined them in a single experiment (Fig. 1B). On mixing fresh blood from normal monkeys with serum from pre-vaccinated monkeys, thrombus formation was observed in all monkeys (Fig. 3). However, a marked delay in thrombus formation was observed on mixing fresh blood and serum from two monkeys (No. 1 and 4). Because the risk of bleeding due to long-term antithrombotic effects is a clinical problem, the bleeding times were checked in two monkeys in No. 3 and 4, and were 300 s and 690 s before vaccination, and 330 s and 540 s after immunization. This indicated that the vaccine did not increase the risk of bleeding. Next, we assessed whether the produced antibody could recognize the epitope, as well as possible anti-thrombotic effects. The post-vaccination serum obtained from No. 4 monkey was mixed with epitope peptide or control peptide, and then added to fresh monkey blood and tested in T-TAS (Fig. 4). The addition of the control peptide showed no thrombus formation, whereas the epitope peptide partially suppressed the anti-thrombotic effects of the vaccinated serum, indicating that the produced antibody could recognize the target epitope in S100A9.

## Discussion

In a previous study, we have reported the efficacy of the anti-S100A9 vaccine in mice; however, the amino acid sequence differs between humans and mice. In the present study, a newly designed peptide vaccine against human and monkey S100A9 demonstrated the successful inhibition of thrombus formation in monkey blood, although some monkeys showed lower anti-thrombotic effects. As similar with our previous study using complete/incomplete Freund’s adjuvant, anti-thrombotic effects were demonstrated in monkeys using aluminum salt Alum.

All monkeys developed anti-S100A9 antibodies, while two monkeys failed to demonstrate anti-thrombotic effects. This result is similar to our previous report, which demonstrated that only half the investigated mice showed anti-thrombotic effects despite antibody production against S100A9^6^. However, the similar pattern of reactions were also observed in clopidogrel treatment^6^ and in other molecules in rodent models, which were examined in various thrombosis models^7–10^. From this perspective, the S100A9 vaccine will be as effective as other anti-thrombotic agents, but there is a possibility that other signals except S100A9/CD36 signal, compensated for the thrombotic reaction in the non-responsive monkeys. One of such compensation might be the thrombospondin-1(TSP-1)/CD36 signal, which was reported to induce platelet activation through inhibition of the cAMP/protein kinase A signaling cascade^11^. Development of combined polypeptide vaccine based on proteins related to thrombosis, such as S100A9 and TSP-1, might improve the effects of the anti-thrombotic vaccine.

Although vaccination is a promising approach to improve medication adherence owing to its long action, safety concerns regarding the long-term bleeding risk should be addressed, although no increase in bleeding time was observed in the present study, as well as the previous study in mice^6^. As idarucizumab was developed for dabigatran reversal, the reversal of anti-thrombotic effects by target epitopes demonstrated possible neutralization in emergent situations such as cerebral hemorrhage. However, the reversal effect of the peptide is partial, possibly because of the rapid enzymatic degradation of the peptide^12^. It is necessary to optimize the epitope peptide to increase the stability in blood for clinical use for reversal of the anti-thrombotic effects.

For S100A9, the reported receptors include RAGE (receptor for advanced glycation endproducts), TLR4 (toll-like receptor 4), and CD36; however, the receptor responsible for platelet activation was revealed as CD36^5^. Although the binding site of S100A9 to CD36 has not been identified, a recent report has shown that 73–85 amino acids in S100A9 specifically interacts with TLR4^13^. Another recent report has revealed that S100A12 inhibited S100A9/RAGE signaling by binding to the residues F48, K50, K51, E56, I59, R85, and L86 of S100A9^14^. These reports suggested that the antibody produced with this vaccine, recognizing 102 to 112 amino acids, might inhibit only S100A9/CD36^5^. As S100A9/TLR4 or S100A9/RAGE signaling is not only involved in inflammatory signaling but is also protective in acute lung injury^15^, the specific inhibition of S100A9/CD36 signaling mediated by this vaccine is an ideal anti-thrombotic strategy without inducing unfavorable side effects.

The limitation of this study is that its efficacy is not demonstrated in human blood. Although we determined an appropriate epitope in monkeys, which is common with that in humans, clinical effectiveness needs to be clarified in human trials as immunological reactions differ between humans and monkeys^16,17^. Second, this study did not demonstrate the efficacy and safety of this vaccine in combination with an antiplatelet agent. As it takes several weeks to achieve sufficient antibody titers to observe anti-thrombotic effects, the combination of the vaccine and antiplatelet agents should be examined in future studies. Third, we did not perform the experiment with alum alone. Because KLH, which strongly enhances Th2 cell immune responses as same as Alum, did not show anti-thrombotic effects in mice^6^, and the anti-thrombotic effects were not reported in Alum when used as an adjuvant in clinical, we suppose the anti-thrombotic effects in the present study were not due to the effect of Alum. Fourth, the vaccinated mice were not tested for anti-thrombotic effect in the present study. The change in amino acid sequence from our previous study^6^ might eliminate the anti-thrombotic effect in the vaccinated mice, but we speculate that the change might not affect the effects in mice due to the similarity of anti-thrombotic effects between the present study and the previous study^6^. Further studies on the differences in anti-thrombotic effects between mice and monkeys will provide information for the future studies regarding an appropriate model system.

In conclusion, the present study demonstrated the feasibility of the S100A9 anti-thrombotic vaccine in monkeys. Although further investigations to optimize the dose and duration of effectiveness, as well as safety, are necessary, this vaccine will be a novel strategy to prevent recurrent stroke attributed to poor medication adherence.

## Methods

### Vaccine design and synthesis

In the present study, 2–11 amino acids (ANKAPSQMER), 97–106 amino acids (ENNPRGHGHS), or 104–113 amino acids (GHSHGKGCGK) in mice S100A9 and 102–111 (GHHHKPGLGE) in human and rhesus monkey were synthesized by Boc solid-phase peptide synthesis and were purified by high-performance liquid chromatography (Peptide Institute Inc., Japan). The peptide was conjugated with keyhole limpet hemocyanin (KLH) at Peptide Institute Inc. Briefly, KLH (Calbiochem, USA) was dissolved in phosphate-buffered saline (pH 8) and stirred gently. Then, N-(6-maleimidocaproyloxy) succinimide (EMCS, DOJINDO LABORATORIES) dissolved in dimethyl sulfoxide was slowly added to the KLH solution and stirred for 3 h at room temperature. The reaction mixture was dialyzed in phosphate-buffered saline containing 10 mM MgSO_4_ several times at 4 °C. Next, antigen peptide dissolved in 6 M guanidinium chloride was added to maleimide-activated KLH. The mixture was stirred at room temperature overnight, and the reaction mixture was dialyzed with H_2_O several times. The peptide-KLH conjugate was lyophilized, and a part of the conjugate was hydrolyzed. The amount of peptide contained in the conjugate was calculated by amino acid analysis.

### Vaccination and bleeding time

All experiments were approved by the Institutional Animal Care and Use Committee of Osaka University (30-041-001) and conducted in accordance with the Osaka University Guidelines, which are based on the National Institutes of Health’s Guide for the Care and Use of Laboratory Animals, and with the ARRIVE guidelines. In the mice experiments, the peptide solutions (400 µg/mL, 50 µL per body) were mixed with an equal volume of TiterMax Gold adjuvant (TiterMax USA, Inc.) and vortexed for 30 min at room temperature before subcutaneous administration via a 23-gauge needle. The vaccine was injected into 9 male C57BL6/J mice (CLEA Japan), aged 8 weeks, twice every 2 weeks. In the monkey experiments, monkeys were trained to become accustomed to people by handing them supplementary food (dried fruit, etc.) during their daily care. We also acclimatized them to be seated in monkey chairs, where the injection of vaccine and collection of blood were performed. The experimenters were also trained to handle monkeys to reduce the stress of capturing, injection, and blood collection. Because this is a non-invasive technique, no anesthesia and analgesics were administered. Four male rhesus monkeys, aged 3 to 4 years, were administered an intradermal injection of 200 µg of the vaccine with Alum (Thermo Scientific, USA) three times at 2 weeks intervals. Blood collection and measurement of bleeding time were performed before and 2 weeks after the last vaccination. Serum was stored at − 80 °C.

Bleeding time was measured following the Duke methods^18^. Briefly, the earlobe was cleaned with alcohol and punctured with a lancet. The blood was blotted every 30 s using a filter paper. The time required for cessation of bleeding was recorded as bleeding time. There were no excluded animals because no abnormal behavior and body weight loss were observed in the present study. All procedures and analyses were conducted by examiners blinded to the experimental conditions.

### Platelet thrombus formation under arterial shear conditions

Platelet thrombus formation was measured by T-TAS, which is an automated microchip-based flow-chamber system developed for easy and rapid assessment of platelet thrombus formation under flow conditions. This system analyzes different thrombus formation processes with a simple procedure, employing two disposable microchips with different thrombogenic surfaces^19^. One chip, the platelet chip (PL-chip), contains 25 capillary channels coated with type I collagen.

Briefly, blood was obtained from male monkeys aged 3 to 4 years and anticoagulated with hirudin. Whole blood (960 µL) was mixed with 240 µL of pre-vaccinated or vaccinated serum. The mixture was maintained at room temperature for 10 min, and then, 330 µL of the mixture was applied to the PL-chip at flow rates of 24 µL/min, corresponding to initial wall shear rates of 2000/s. The thrombus formation processes inside the tip were analyzed by continuous monitoring of the flow pressure changes resulting from capillary occlusion. The area under the flow pressure curve (AUC) was computed to assess platelet thrombogenicity inside the microchips.

To assess the reversal effects of the epitope peptide, epitope peptide (GHHHKPGLGE, 102–111 aa) or control peptide (CPNTEAKDFL) was added to the mixture of whole blood and vaccinated serum.

### Enzyme-linked immunosorbent assay (ELISA)

The S100A9 antibody responses were measured by coating ELISA plates with 10 µg/mL each S100A9 peptide conjugated to bovine serum albumin (Peptide Institute Inc., Japan) in carbonate buffer overnight at 4 °C. After blocking with 5% skim milk solution in phosphate-buffered saline, serum samples were diluted in 5% skim milk, followed by overnight incubation at 4 °C. Detection was performed using goat anti-IgG horseradish peroxidase conjugate (1:10,000, Abcam, UK), followed by the application of TMB solutions. The half-maximal antibody titer was determined according to the highest value in the dilution range of each sample.

### Western blot analysis

We examined whether the induced antibodies could recognize and bind to the recombinant S100A9 proteins. Recombinant human S100A9 (5 µg, R&D, USA) were separated by SDS-PAGE and blotted onto polyvinylidene difluoride membranes (Merck KGaA, Germany). The membrane was cut into 4 pieces, each of which were separately incubated with skim milk (2 mL), including serum from pre-immunized or immunized monkeys (20 µL), rabbit anti-human S100A9 as a positive control (2 µg, Abcam) or isotype IgG-control purified from monkey (20 µg, Peptide Institute Inc.). Each membrane was incubated with HRP-conjugated antibodies specific for monkey IgG (1:10,000, Nordic**-**MUbio/IgG(H + L)/PO, Netherlands) for the serum from monkeys and the isotype IgG control, or rabbit IgG (1:1000, GE Healthcare, UK) for anti-human S100A9. Chemiluminescence signal for these membranes was detected and analyzed with ChemiDoc Touch (Bio-Rad, USA).

### Statistical analysis

Data are expressed as mean ± standard error of the mean (SEM). Data were analyzed using GraphPad Prism version 8 (GraphPad, USA). Between-group differences were assessed using Mann–Whitney test (Fig. 3B). A value of p < 0.05 was considered statistically significant.

## Supplementary Information

Supplementary Information.

## Data Availability

The data that support the findings of this study are available from the corresponding author upon reasonable request.
