# Supplementary material for: Development of anti-thrombotic vaccine against human S100A9 in rhesus monkey
Source: Sci Rep. 2021 Jun 1;11:11472. doi: 10.1038/s41598-021-91153-y (PMC8169762; doi:10.1038/s41598-021-91153-y)
Supplement: Supplementary file 1 — Supplementary Information. [file 41598_2021_91153_MOESM1_ESM.docx]

**Development of Anti-Thrombotic Vaccine Against Human S100A9 in Rhesus Monkey**

Munehisa Shimamura^1,2*^, Koichi Kaikita^3^, Hironori Nakagami^2^, Tomohiro Kawano^1,2^, Nan Ju^2^, Hiroki Hayashi^2^, Ryo Nakamaru^2^, Shota Yoshida^2^, Tsutomu Sasaki^1^, Hideki Mochizuki^1^, Kenichi Tsujita^3^, Ryuichi Morishita^4*^

^1^Department of Neurology, Graduate School of Medicine, Osaka University

^2^Department of Health Development and Medicine, Graduate School of Medicine, Osaka University

^3^Department of Cardiovascular Medicine, Graduate School of Medical Sciences, Kumamoto University

^4^Department of Clinical Gene Therapy, Graduate School of Medicine, Osaka University

***Corresponding authors**: Ryuichi Morishita^1^, Munehisa Shimamura^2,3^

^1^Department of Clinical Gene Therapy, ^2^Department of Health Development and Medicine and ^3^Department of Neurology, Osaka University Graduate School of Medicine, Center of Medical Innovation and Translational Research (6th floor, Room 0612B), Osaka University, 2-2 Yamada-oka, Suita, Osaka 565-0871, Japan

Tel: +81-6-6210-8359; Fax: +81-6-6210-8360

Email: morishit@cgt.med.osaka-u.ac.jp; shimamuu@cgt.med.osaka-u.ac.jp

**Supplementary Figure S1**


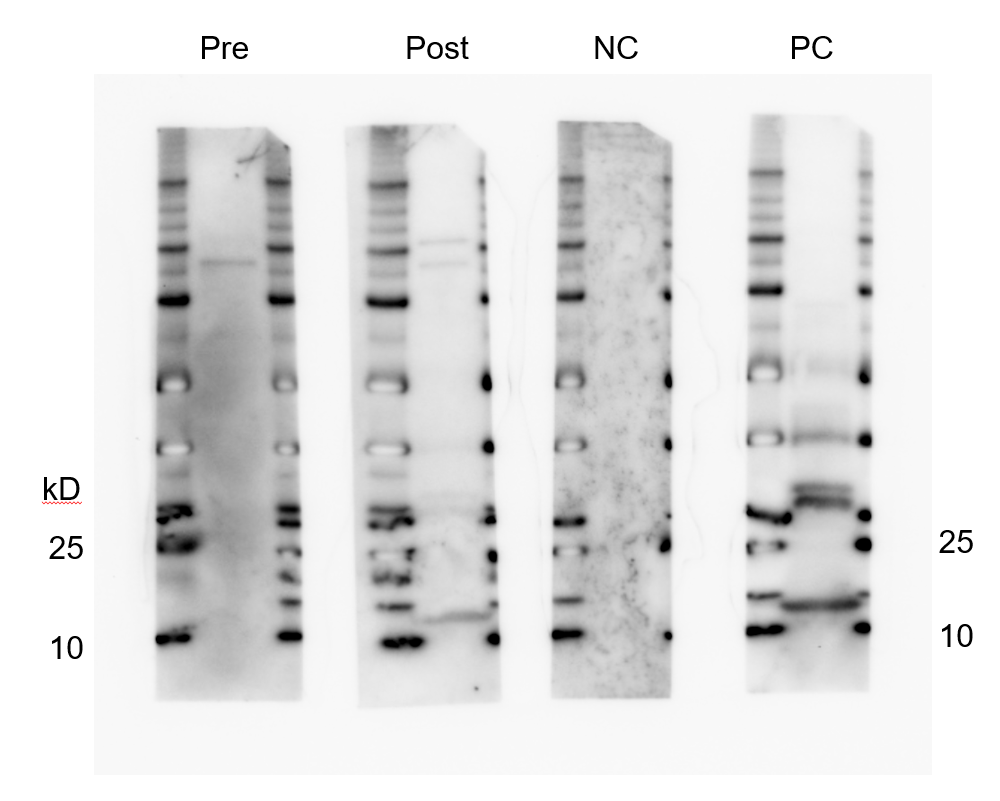


Full length gels and blots in western blot analysis for production of antibodies specific for recombinant human S100A9 protein (14 kDa) in Fig. 2C. Pre, the serum samples from pre-vaccination; Post, the serum samples from post-vaccination; NC, IgG purified from another normal monkey; PC, the commercially available anti-S100A9 antibody.
